# Performance of Three Isolates of *Metarhizium anisopliae* and Their Virulence against *Zeugodacus cucurbitae* under Different Temperature Regimes, with Global Extrapolation of Their Efficiency

**DOI:** 10.3390/insects10090270

**Published:** 2019-08-26

**Authors:** Susan K. Onsongo, Bernard M. Gichimu, Komivi S. Akutse, Thomas Dubois, Samira A. Mohamed

**Affiliations:** 1 Plant Health Division, International Centre of Insect Physiology and Ecology (icipe), Nairobi 00100, Kenya; 2 Department of Agricultural Resource Management, University of Embu, Embu 60100, Kenya

**Keywords:** entomopathogenic fungus, germination, melon fly, percentage mortality, radial growth, sporulation

## Abstract

The performance of entomopathogenic fungi in pest control is usually affected by both biotic and abiotic factors. This study aimed to determine the effects of various temperatures (15, 20, 25 and 30 °C) on conidial germination, mycelial growth and conidial density and virulence to the melon fly *Zeugodacus cucurbitae* of three selected isolates of *Metarhizium anisopliae.* The three isolates, ICIPE 18, ICIPE 30 and ICIPE 69, had previously been selected in laboratory bioassays. Percentage mortality by the three isolates ranged between 16.25% and 100.0% across the different temperatures. The isolates ICIPE 69 and ICIPE 18 recorded the highest percentage mortality of 96.25% and 100% and the shortest LT_50_ values of 2.61 and 2.63 days, respectively, at 30 °C. However, at 30 °C, ICIPE 69 produced the highest number of conidia of 90.5 × 10^7^ /mL and was therefore selected for global mapping to predict its efficacy against *Z. cucurbitae* using the geospatial temperature data layer and the best fitted quadratic model. The map showed that the isolate would be more effective in the tropics than in temperate climates.

## 1. Introduction

Cucurbits are widely cultivated around the world and are among the most important fruits and vegetables consumed in Africa [1]. They are a key source of income for small scale farmers [2] and also very rich in several vitamins and minerals [1]. In Kenya, cucurbits are widely cultivated and among the most consumed commodities [3]. However, their production has declined over the years owing to several constraints, especially insect pests and diseases [2]. Among the major insect pests of cucurbits are fruit flies (Diptera: Tephritidae), aphids (Hemiptera: Aphididae), greenhouse whitefly *Trialeurodes vaporariorum* Westwood (Hemiptera: Aleyrodidae), thrips (Thysanoptera: Thripidae) spider mites *Tetranychus urticae* Koch (Acari: Tetranychidae) and beetles *Epilachna* spp. (Coleoptera: Coccinellidae) [4,5]. The diseases of economic importance include watermelon mosaic virus (WMV, *potyvirus*), angular leaf spot *Xanthomonas fragariae* (Xanthomonadales: Xanthomonadaceae) and damping off *Pythium* spp (Peronosporales: Pythiaceae) [6,7,8].

Globally, Tephritid fruit flies are the most serious insect pests of both fruits and vegetables [9,10]. In Africa, member of the genus *Dacus* (native to Africa) have been the most dominant and damaging fruit flies resulting in significant yield losses [11]. However, the problem was further compounded by the widespread invasion of the alien invasive species *Zeugodacus cucurbitae* (Coquillett) (Diptera: Tephritidae) (formerly known as *Bactrocera cucurbitae*), very often leading to total crop failure [12,13] due to the pest’s is high fecundity [13] and the lack of natural enemies on the continent. Moreover, *Z. cucurbitae* is highly polyphagous posing a serious threat for production of non-cucurbitaeous crops [14].

Tephritid fruit flies cause direct losses through oviposition by the female fly under the fruit skins and feeding of emerged larvae, causing fruit rot [15]. A study carried in Kenya showed that 66.8% of the losses on bittergourd *Momordica charantia* L. (Cucurbitales: Cucurbitaceae) were solely due to fruit fly infestation [16]. Furthermore, the pest causes considerable indirect losses due to stringent quarantine restrictions for export imposed by importing countries [17].

Most farmers in the world use synthetic chemical insecticides for management of *Z. cucurbitae* [18], which are detrimental to human and environmental health and whose excessive use results in pest resistance [19]. For example, a population of *Z. cucurbitae* obtained from Hainan Island showed resistance to some classes of chemical insecticides [20]. Biological control options using entomopathogenic fungi are being researched on as alternatives to chemical pesticides [21]. Recently, we screened 15 isolates of *Metarhizium anisopliae* (Metschnikoff) Sorokin (Hypocreales: Clavicipitaceae) and *Beauveria bassiana* (Balsamo) Vuillemin (Hypocreales: Cordycipitaceae) against adult *Z. cucurbitae* and selected 3 strains of *M. anisopliae*, ICIPE 18, ICIPE 30 and ICIPE 69, due to their high virulence [22]. 

However, studies have shown that effectiveness of these entomopathogenic fungi is affected by both abiotic and biotic factors [23], with temperature being among the key factors [23]. Several, studies have reported varying effects of temperature on germination, growth rate, sporulation, survival and host-pathogen interaction of entomopathogenic fungi with the optimum temperature for most of them ranging between 20–30 °C, depending on the isolate [24,25,26,27]. Therefore, for optimal field application, the most potent isolates should also be tolerant to temperature ranges where the target pest is dominant or/and have a wider thermal tolerance range. Development and active populations of most *Bactrocera* spp. can be found at temperatures ranging from 25–30 °C [28,29]. For example, a study by Dimbi et al. [30] selected *M. anisopliae* isolate ICIPE 20 as the most suitable entomopathogenic fungal isolate for the management of three species of Tephritid fruit flies based on its high growth rate, activity over a broader range of temperatures and high virulence at the optimal temperature.

The present study aimed at determining the effects of various temperatures on conidial germination, mycelial growth rate and sporulation of the three selected isolates of *M. anisopliae* and their virulence against *Z. cucurbitae*. A global map was also projected to show the efficiency of the selected isolate. The findings of this study will guide the selection of the best isolates for management of *Z. cucurbitae* at different ecologies.

## 2. Materials and Methods

### 2.1. Rearing of Z. cucurbitae

Adult *Z*. *cucurbitae* were obtained from the mass rearing unit at the International Centre of Insect Physiology and Ecology (*icipe*, Nairobi, Kenya). They were exposed to butternuts *Cucurbita moschata* Duch. (Cucurbits: Cucurbitales) for 24–48 h for oviposition. A plastic container (35 × 20 × 12 cm) was filled with sterile sand up to a depth of 2 cm and fitted with a wire mesh at 5 cm above the sand inside the container. The wire mesh was used to hold the infested butternut to allow the mature larvae to pop out and drop into the sand to pupate. The pupae were collected in 90 mm diameter Petri dishes and placed in Perspex cages (15 × 15 × 15 cm) for adults to emerge. Emerged adult flies were maintained on a sugar and yeast hydrolysate based artificial diet [31] at a relative humidity of 45%, temperature of 27 ± 2 °C and photoperiod of 12 h of light and 12 h of darkness (L12: D12) according to Dimbi et al. [30].

### 2.2. Fungal Isolates

Three fungal isolates of *M. anisopliae*, ICIPE 18, ICIPE 30 and ICIPE 69, were selected after laboratory screening of 15 isolates based on their performance against *Z cucurbitae* (data not published). ICIPE 18 was obtained from African Stem Borer *Busseola fusca* Fuller (Lepidoptera: Noctuidae) in Kendubay, Kenya, ICIPE 30 from a soil sample from Mbita, Kenya and ICIPE 69 from a soil sample from Matete, Democratic Republic of Congo. All isolates were preserved at −80 °C at *icipe* prior to use.

Prior to the bioassays, the isolates were revived on Sabouraud dextrose Agar (SDA) media in 90 mm diameter Petri dishes and incubated at 25 ± 2 °C for 21 days in the laboratory for sporulation. After 21 days, conidia were harvested by scraping the surface and suspended in 10 mL of sterile 0.01% Triton water in a 30 mL universal bottle containing 3 mm diameter glass beads to obtain a stock solution according to Tumuhaise et al. [27]. A homogeneous suspension of conidia was obtained by vortexing for 3 min at 700 rpm, and a final concentration prepared by diluting from the stock. The conidial suspension was quantified and adjusted to 3 × 10^6^ conidia/mL using a hemocytometer under a light microscope (LEICA DM 2000, Leica Microsystems, Morrisville, NC, USA) at magnification of ×40.

### 2.3. Effect of Temperature on Conidial Germination

For each isolate (ICIPE 18, ICIPE 30 and ICIPE 69), 0.1 mL conidial suspension with a concentration of 3 × 10^6^ conidia/mL was spread on 90 mm diameter Petri dishes containing SDA. The plates were sealed with parafilm and incubated at 15, 20, 25 and 30 °C in complete darkness for 16–18 h. Subsequently, conidial germination was halted by spreading 1 mL formaldehyde (0.5%) per plate, and three sterile microscope cover slips were placed randomly on the surface of each inoculated plate. Viability of each fungal isolate was determined by randomly selecting a total number of 100 conidia and counting both germinated and non-germinated conidia beneath each coverslip under a light microscope at ×40 magnification, and mean percentage germination was determined. Four replicates were used.

### 2.4. Effect of Temperatures on Sporulation

For each isolate, Petri dishes containing SDA were inoculated as described in Section 2.3 and allowed to grow for three days to obtain mycelial mats. Plugs (ca. 5 mm) of mycelium were cut from the plates using an 8 mm-diameter cork borer and placed upside down at the center of a 90 mm Petri dish. They were then incubated at 15, 20, 25 and 30 °C in darkness for 16–18 h. Conidia were harvested from 5 mm diameter mycelial discs into 10 mL triton water in a universal bottle, which was vortexed for 3 min to obtain a homogenous solution. The conidia suspension with a concentration of 1 × 10^7^ was prepared then conidia were counted under a light microscope (40×) using a Neubauer hemocytometer and expressed as conidial density. Each treatment had four replicates.

### 2.5. Effect of Temperature on Radial Growth

For each isolate, mycelial mats. Plugs (ca. 5 mm) were obtained as described in Section 2.4 and incubated at 15, 20, 25 and 30 °C in darkness for 16–18 h. After inoculation, mycelial radial growth was measured daily for 12 days using two cardinal diameters drawn on the bottom of each plate and recorded. Four replicates were used for each treatment.

### 2.6. Effect of Temperature on Fungal Virulence

**Figure 1:**
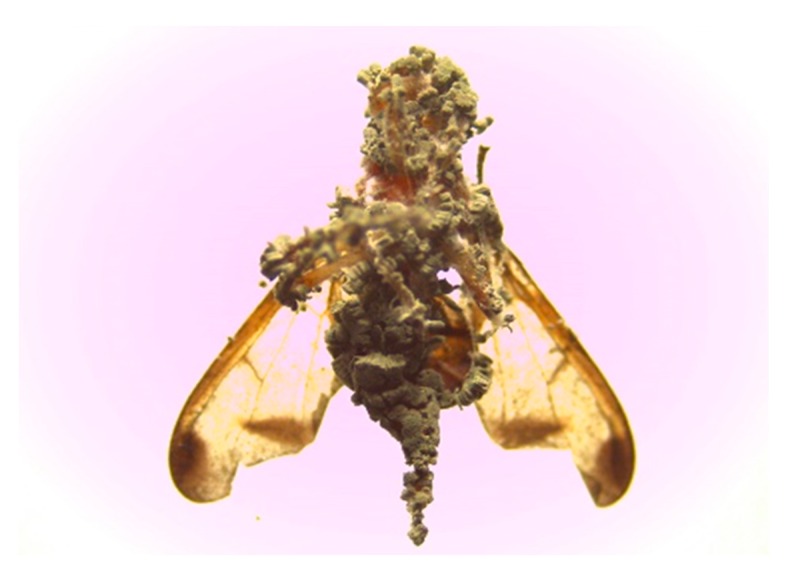
A mycosed adult melon fly (*Zaugodacus cucurbitae)* due to *Metarhizium anisopliae*.

While under laminar flow, conidia were scrapped from 21-day-old cultures using sterile wire loop. A mass of 0.3 g dry conidia of each fungal isolate was weighed, and a spatula was used to evenly spread the conidia onto the velvet inside a 9.5 cm × 4.8 cm plastic vial, which acted as the contaminating device. Twenty adult flies (5–7 day-old) were randomly picked from the insect colony and introduced into the contaminated device for 5 min to walk on the velvet material, while uninoculated insects acted as a control and were exposed to contamination devices that had not been inoculated [30]. After 5 min, the twenty flies in each replicate from both infected insects and control insects were separately transferred into clean Perspex cages (15 cm × 15 cm × 15 cm) and provided with a moistened cotton bud as a source of water and sugar and yeast hydrolysate based artificial diet as food in a 90 mm diameter Petri dish. All the treatments were kept at 15, 20, 25 and 30 °C, and fly mortality was monitored daily for 4 days. Dead flies were picked daily, surface-sterilized in 70% EtOH and 2.5% NaOH for 2–3 min, rinsed thrice in sterile distilled water and transferred into 90 mm diameter Petri dishes lined with damp sterilized filter paper to allow fungal growth on the surface of the cadaver. Mycosis was confirmed by examining the surface of the cadaver under a light microscope, and mortality due to *M. anisopliae* infection was confirmed by the presence of green conidia on the surface of the cadaver (Figure 1).

### 2.7. Data Analysis

Percentage mortality of adult flies was corrected for natural mortality using Abbott’s formula [32] and checked for normality using the Shapiro–Wilk test. The data were angular transformed prior to two-way analysis of variance (ANOVA) using binomial regression analysis. Separation of means was carried out using the Tukey HSD test. Lethal time to 50% and 90% mortality (LT_50_ and LT_90_) values was calculated using generalized linear model (GLM) using the function “dose.p” from the MASS library [33]. Percentage germination of fungal isolates and conidial density were checked for normality, then subjected to two-way analysis of variance ANOVA after angular transformation and means separated using Tukey HSD test. Conidial radial growth rates were fitted by regression analysis (y = mx + c) and then the linear regression slope (m), which indicates the growth rates (velocity in mm d^−1^), used as the main parameter to evaluate the influence of temperature on fungal growth [34,35,36,37,38].

Temperature-based modelling was performed for each isolate to determine the optimum, minimum and maximum temperatures for virulence against *Z. cucurbitae*. The relationship between temperature and mean percentage mortality was obtained by fitting the data to nonlinear models. After testing on 13 different equations available in “easynls” package [39], a quadratic expression m(T) = b_0_ + (b_1_ × x) + (b_2_ × x^2^) (with m = predicted mean mortality of *Z. cucurbitae* in relation to temperature T, x = variable temperature, and b_0_, b_1_ and b_2_ = estimated parameters) had the lowest Akaike information criterion (AIC) estimate and therefore selected as the best fit to the experimental data [23]. All data analyses were performed using R (version 3.2.5) statistical software packages (R Development Core Team [40]).

Global suitability of *M. anisopliae* isolates in controlling *Z. cucurbitae* was tested using the geospatial temperature data layer and the best fitted quadratic model. The maximum temperature (°C) (t_max_ 30 s) was downloaded from http://worldclim.org/version2. The data are available at spatial resolutions of 30 s (~1 km^2^) for the entire globe. The data were imported into ArcGIS v10.3.1 (ESRI, Redlands, CA, USA) for further analysis. The raster calculator function in spatial analyst toolbox was used to derive global fungi use suitability maps using the temperature and mortality equation y = −0.41x^2^ + 28.81x − 249.29 (y = percentage of mortality caused by the fungi and x = temperature). The global temperature dataset was used as pixel-based representative of temperature at 1 km spatial resolution.

## 3. Results

### 3.1. Effect of Temperature on Conidial Germination

Conidia of the three selected isolates of *M. anisopliae* germinated at all the temperatures tested with mean percentage germination ranging from 2.90% to 98.96% (Table 1). The conidial percentage germination for all the three isolates was significantly affected by the various temperature regimes but was not affected by isolate (Table 1). However, there was no interaction between fungal isolate and temperature regime (Table 1). The highest conidial percentage germinations were recorded at 25 °C and 30 °C, which were not significantly different from each other but were significantly different from the lower temperature regimes of 15 °C and 20 °C for all the isolates. The optimal temperature for conidial percentage germination was therefore observed to be between for 25 °C and 30 °C for all the three isolates (Table 1).

### 3.2. Effect of Temperature on Radial Growth

The temperature and isolate were found to have a significant effect on fungal growth rate. The growth rate increased with temperature with the highest growth being recorded at 30 °C and the lowest at 15 °C (Table 2). There was no interaction between the temperature and the fungal isolates with regard to fungal growth rate.

### 3.3. Effect of Temperature on Conidial Density

**Figure 2:**
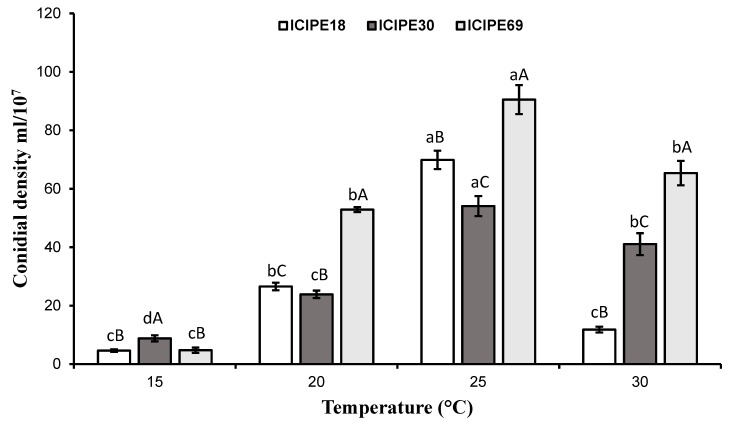
Effect of temperature on conidia production/sporulation of the three *Metarhizium anisopliae* isolates. Means with the same lowercase letter are not significantly different under same temperature among different isolates while those with the same upper case letter are not significantly different under same isolate across different temperature based on Tukey’s HSD multiple range test at *p* = 0. 05.

Sporulation of the three fungal isolates was significantly affected by temperature (*F* = 305.2; df 3,36; *p* < 0.001) and isolate (*F* = 104.4; df 3,36; *p* < 0.001). There was also an interaction between temperature and isolate (*F* = 27.3; df 3,36; *p* < 0.001). The isolate ICIPE 69 produced more conidia spores at 20 °C, 25 °C and 30 °C, while ICIPE 18 and ICIPE 30 had the highest conidial density at 25 °C. The best sporulation temperature for all the isolates was found to be at 25 °C (Figure 2).

### 3.4. Effect of Temperature on Virulence of M. anisopliae Isolates to Z. cucurbitae

All the three isolates were virulent against *Z cucurbitae*, with their percentage mortality increasing from 31.25%–100.0%, 16.25%–80.0% and 23.75%–96.25% for ICIPE 18, ICIPE 30 and ICIPE 69, respectively, across the temperature regimes of 15–30 °C. Temperature and isolate were found to significantly have an effect on percentage mortality. The highest mortality occurred at 25 °C and 30 °C temperature regimes, which were not significantly different from each other (Table 3). Irrespective of temperature, the highest percentage mortality was caused by isolate ICIPE 18 followed by ICIPE 69. There was a significant interaction between the temperature and the fungal isolates (*F* = 2.63; df = 6,36; *p* < 0.05) on percentage mortality.

The efficacy was also measured by the lethal time to 50% and 90% mortality (LT_50_ and LT_90_) values. The shortest LT_50_ and LT_90_ values were observed at 30 °C for all the three isolates (Table 4). Therefore, isolates ICIPE 18 and ICIPE 69 portrayed the highest efficacy in the control of *Z. cucurbitae* within the temperature range of 25 to 30 °C.

**Figure 3:**
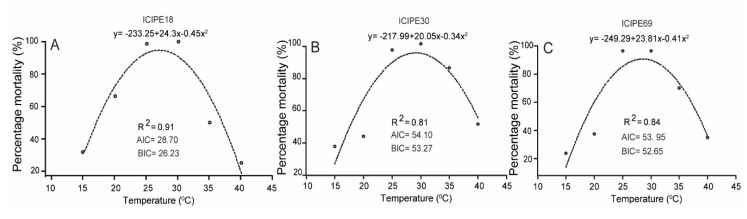
Temperature-dependent percentage mortality rates of adult *Z. cucurbitae*. ICIPE 18 (**A**), ICIPE 30 (**B**) and ICIPE 69 (**C**). Markers are observed mean mortalities. AIC is Akaike information criterion and BIC is Bayesian information criterion.

A nonlinear regression model was used to predict the efficacy of fungi in relation to temperature. In all the isolates tested, the quadratic model indicated that percentage mortality of *Z. cucurbitae* increased significantly as temperature increased up to an optimum temperature ranging between 20 °C and 35 °C, beyond which the percentage mortality reduced (Figure 3). The model predicted the minimum temperatures to range between 10 °C and 15 °C and the maximum to be between 40 °C and 44 °C.

**Figure 4:**
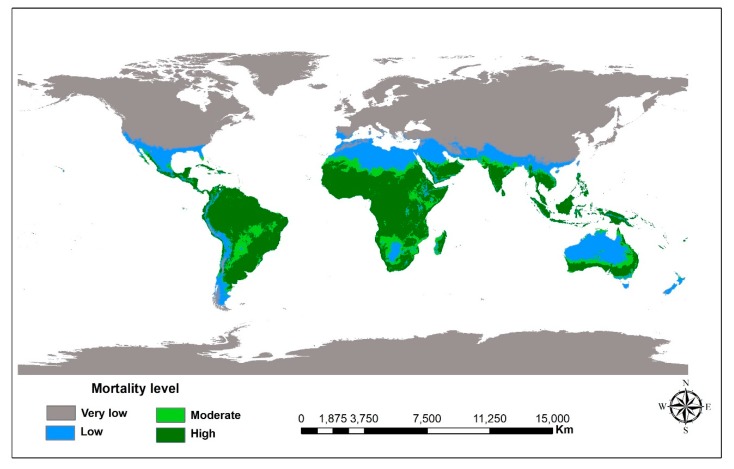
Global map predicting the efficacy of *M. anisopliae* isolate ICIPE 69 against *Z. cucurbitae* using the geospatial temperature data layer and the best fitted quadratic model.

From the three isolates, the best candidate from the activities was used for prediction. The global prediction of percentage mortality for ICIPE 69 is shown in Figure 4. This is relevant to show which areas the fungus would be more effective in controlling *Z. cucurbitae*. Four colors were used to indicate the strength of the prediction. The map shows that the fungus would be most effective in the tropical climates of Africa and South America and least effective in Asia, Canada and United States of America.

## 4. Discussion

In entomopathogenic fungi, the positive association of the speed of conidial germination and radial growth with fungal virulence has been well documented [41,42,43,44]. For instance Shah et al. [42] and Andersen et al. [43] working with *M. anisopliae* reported a positive correlation between the germination speed and the virulence. Another, key trait of the efficacy of the entomopathogenic fungi is the rate of sporulation as it results in more of the target pest infection. All these traits (germination, radial growth and sporulation) are reported to be influenced by temperature. *M. anisopliae* isolates have proven to be very pathogenic to *Z. cucurbitae* [22]. This study further evaluated the performance of these isolates under different temperatures.

Although all isolates had performed comparably in term of spore germination at all temperatures tested in this study, spore germination of the three isolates was affected by temperature, with the optimum temperature being 25–30 °C. In a related study, Bayissa et al. [38] and Bugeme et al. [45] reported similar optimal temperature range for some isolates of *M. anisopliae* and *B. bassiana*, respectively. Likewise, the same temperature range was found to be optimal for spore germination of fungi such as such as *Penicilium expansum* (Link) Thom. (Eurotiales: Trichocomaceae) [46] and *Aspergillus ochraceus* Wilh. (Eurotiales: Trichocomaceae) [47]. This was attributed to the fact that the three isolates originated from location with similar environmental conditions.

For fungal radial growth, both temperature and isolates had a significant effect. The differential radial growth with temperature recorded in this study concurs with that reported by Tumuhaise et al. [27] and Bayissa et al. [38] for *M. anisopliae* isolates. Therefore, the optimal temperature for radial growth of these isolates was 30 °C. This was within the optimum range of 20 to 30 °C reported in other entomopathogenic studies [35]. The highest growth rate observed among the isolates ranged from 3.79 to 4.08, which was within the range observed by Fargues et al. [34] on different isolates of *M. anisopliae* and by Cabanillas and Jones [48] on most *Isaria* isolates (Hypocreales: Clavicipitaceae). However, these values were higher than Dimbi et al. [30] and lower than Bayissa et al. [38] in their studies. Although the isolates were obtained from similar conditions, the difference in their radial growth could mean that fungal isolates behave differently after a longer exposure to different temperatures.

Fungal spore density was influenced by both temperature and isolates. The optimum sporulation temperature was found to be 25 °C with a significant reduction of conidia at 30 °C. This finding concurs with those by Tefera and Pringle [24], Arthurs and Thomas [49] and Borisade and Magan [50] for other isolates of *M. anisopliae* and *B. bassiana*. Likewise, a study by Chauvet and Suberkropp [51] on aquatic Hyphomycetes showed that *Lunulospora curvula* Ingold and *Tetracladium marchalianum* DeWild. sporulated at an optimum temperature of 25 °C, which is in agreement with our results. Moreover, the optimal temperature for the sporulation of the endophytic fungus *Colletotrichum* spp (Glomerellales: Glomerellaceae) was reported to be within the same range with that of this study [52]. In this study, optimum sporulation temperature was found to be lower than that of radial growth. This could mean that the sporulation of the selected isolates is heat sensitive and hence the sporulation started to decrease with increase of temperature after 25 °C.

The tested *M. anisopliae* isolates had a differential virulence against *Z. cucurbitae*, which also varied with temperature for all the isolates being higher at higher temperatures (25 °C and 30 °C), with isolates ICIPE 18 and 69 outperforming ICIPE 30 in term of *Z. cucurbitae* percentage mortality at these temperatures. This could mean that at optimum temperatures the fungi colonize and establish faster than in lower temperatures. Other studies that reported similar results with *M. anisopliae* isolates on other insects include Yeo et al. [36] on aphid species, Ugine [25] on *Lygus lineolaris* (Palisot de Beauvois) (Hemiptera: Miridae) and Mishra et al. [26] on *Musca domestica* L. (Diptera: Muscidae ). Moreover, a study by Bayissa et al. [38] found out that *M. anisopliae* isolates were more virulent to aphid species at 25 °C and 30 °C than at 15 °C and 20 °C, which was in agreement with this study.

In addition to higher percentage mortality caused by the isolates ICIPE 18 and 69, these isolates have the shortest lethal time at the optimal virulence range of 25 to 30 °C. This finding has a significant implication for management of *Z. cucurbitae*, being generally a low land pest with optimum development temperature range of 25 to 32 °C [29].

Modeling can provide better understanding of thermal tolerance of the biocontrol agents such as biopesticide [53]. In this study, the nonlinear regression model was used to predict the efficacy of fungi in relation to temperature. The minimum, optimum and maximum threshold temperatures for the three tested *M. anisopliae* fungal isolates were estimated by the quadratic equation to be between 10 and 15 °C, 25 and 30 °C and 40 and 44 °C, respectively. These estimates were within the range of values obtained by Rangel et al. [54] on *Metarhizium* spp.

Although the performance of ICIPE 18 and 69 in terms of percentage germination, radial growth and percentage mortality is comparable at optimum temperatures of 25 to 30 °C, ICIPE 69 was more superior with regard to conidia density (sporulation) as well as LT_50_ and LT_90_. Based on this, the efficacy of this isolates against *Z. cucurbitae* was predicted on global scale using the geospatial temperature data layer and the best fitted quadratic model. This result showed that ICIPE 69 would be more effective in the tropics than the temperate regions, which is in agreement with Tumuhaise [55] on first instar larvae of *Maruca vitrata* Fabricius (Lepidoptera: Pyralidae). This isolate could be therefore integrated with other control agents such as use of cue–lure pheromone food bait Street et al. [56] and other cultural practices [18,57]. However, *M. anisopliae* has been found to act differently on different genera or species of non-target pests [58,59]. For example, effects of *M. anisopliae* on searching, feeding and predation by *Orius albidipennis* Reuter (Hemiptera: Anthocoridae) showed that the presence of the *M. anisopliae* increases the searching time and decreases feeding time and predation. It was also able to detect and avoid treated patches [60]. Moreover, there have been successful applications of fungus in the field for management of different pests [61,62,63,64].

This finding has a significant implication for management of *Z. cucurbitae*, generally a low-land pest with an optimum development temperature range of 25 to 32 °C [26]. It is also important to note that ICIPE 69 has been commercialized and registered on other horticulture insect pests (Whiteflies, Mealy bugs and Thrips), and it only requires extension of label to be registered for the management of *Z. cucurbitae* as an effective biopesticide.

## 5. Conclusions

The performance of three evaluated isolates of *M. anisopliae* in terms of conidial percentage germination, sporulation, radial growth and virulence was influenced by temperature, with the optimal efficiency for all isolates being at 25 to 30 °C. Although all isolates showed varied degree of pathogenicity against *Z. cucurbitae*, ICIPE 69 was considered to be the most promising candidate as biopesticide for management of this pest, considering the fact that it had a higher conidia production (sporulation) as well as LT_50_ and LT_90_ values.

## Figures and Tables

**Table 1:** Percentage germination of *Metarhizium anisopliae* isolates at different temperatures.

Temperature	Fungal Isolates		
	ICIPE 18	ICIPE 30	ICIPE 69
15 °C	4.26 ± 0.35 ^cA^	3.65 ± 0.20 ^cAB^	2.90 ± 0.28 ^cB^
20 °C	69.83 ± 1.82 ^bA^	71.74 ± 1.27 ^bA^	71.75 ± 2.99 ^bA^
25 °C	98.86 ± 0.48 ^aA^	97.64 ± 0.45 ^aA^	98.96 ± 0.49 ^aA^
30 °C	98.00 ± 0.23 ^aA^	97.69 ± 0.41 ^aA^	98.56 ± 0.27 ^aA^
Temperature	*F*_3,36_ = 3084.63	*p* < 0.001	
Isolate	*F*_2,36_ = 1.23	*p* = 0.304	
Temperature x isolate	*F*_6,36_ = 1.57	*p* = 0.184	

Means with the same lowercase letter within the column are not significantly different while those with the same uppercase letter within the row are not significantly different based on Tukey’s HSD multiple range test at *p* = 0.05.

**Table 2:** Effect of temperature on the radial growth rates day^−1^ of *M. anisopliae* isolates.

	Fungal Isolates		
Temperature	ICIPE 18	ICIPE 30	ICIPE 69
15 °C	1.32 ± 0.10 ^dA^	0.18 ± 0.07 ^dB^	1.0 ± 0.06 ^dB^
20 °C	2.48 ± 0.11 ^cA^	1.65 ± 0.13 ^cB^	2.44 ± 0.15 ^cA^
25 °C	3.15 ± 0.16 ^bA^	2.85 ± 0.04 ^bA^	3.34 ± 0.13 ^bA^
30 °C	3.88 ± 0.18 ^aA^	3.79 ± 0.15 ^aA^	4.08 ± 0.24 ^aA^
Temperature	*F*_3,36_ = 241.712	*p* < 0.001	
Isolate	*F*_2,36_ = 13.267	*p* < 0.001	
Temperature x isolate	*F*_6,36_ = 2.006	*p* = 0.0904	

Means with the same lowercase letter within the column are not significantly different at a similar temperature while those with the same uppercase letter within the row are not significantly different at different temperatures based on Tukey’s HSD multiple range test at *p* = 0. 05.

**Table 3:** Percentage mortality of adult *Z. cucurbitae* caused by *M. anisopliae* isolates at different temperature regimes at 4 days’ post-exposure.

Temperature	Fungal Isolates		
	ICIPE 18	ICIPE 30	ICIPE 69
15 °C	31.25 ± 3.15 ^cA^	16.25 ± 3.15 ^bA^	23.75 ± 5.15 ^bA^
20 °C	66.25 ± 3.75 ^bA^	22.5 ± 3.23 ^bB^	37.5 ± 8.29 ^bB^
25 °C	98.75 ± 1.25 ^aA^	76.25 ± 3.15 ^aB^	96.25 ± 1.25 ^aA^
30 °C	100.00 ± 0.00 ^aA^	80.00 ± 2.04 ^aB^	96.25 ± 2.39 ^aA^
Temperature	*F*_3,36_ = 214.76	*p* < 0.001	
isolate	*F*_2,36_ = 56.46	*p* < 0.001	
Temperature x isolate	*F*_6,36_ = 2.63	*p* < 0.05	

Means with the same lowercase letter within the column are not significantly different while those with the same uppercase letter within the row are not significantly different based on Tukey’s HSD multiple range test at *p* = 0. 05.

**Table 4:** Lethal time to 50% and 90% mortality of adult *Z. cucurbitae* caused by *M. anisopliae* isolates at different temperature regimes at 95% fiducial limit.

	ICIPE 18		ICIPE 30		ICIPE 69	
Temperature	LT_50_ (days)	LT_90_ (days)	LT_50_ (days)	LT_90_ (days)	LT_50_ (days)	LT_90_ (days)
15 °C	4.89	7.41	5.25	7.00	5.40	8.04
	(4.72–5.06)	(7.04–7.78)	(5.04–5.46)	(6.61–7.40)	(5.19–5.64)	(7.56–8.52)
20 °C	3.36	5.07	5.32	7.69	4.46	6.53
	(3.3–3.41)	(4.95–5.19)	(5.10–5.54)	(7.26–8.13)	(4.34–4.58)	(6.28–6.78)
25 °C	2.71	3.84	2.99	4.70	2.71	3.92
	(2.68–2.75)	(3.78–3.9)	(2.94–3.04)	(4.59–4.8)	(2.67–2.75)	(3.85–3.98)
30 °C	2.63	3.72	2.99	4.54	2.61	3.70
	(2.59–2.66)	(3.66–3.77)	(2.94–3.03)	(4.45–4.63)	(2.57–2.64)	(3.65–3.76)

The values show LT_50_ and LT_90_ in days; values in brackets represent fiducially limit at 95%.
